# Real-World Efficacy and Safety of Bevacizumab in the First-Line Treatment of Metastatic Cervical Cancer: A Cohort Study in the Total Population of Croatian Patients

**DOI:** 10.1155/2021/2815623

**Published:** 2021-08-05

**Authors:** Dora Čerina, Višnja Matković, Kristina Katić, Ingrid Belac Lovasić, Robert Šeparović, Ivana Canjko, Blanka Jakšić, Branka Petrić-Miše, Žarko Bajić, Marijo Boban, Eduard Vrdoljak

**Affiliations:** ^1^Department of Oncology, University Hospital Center Split, School of Medicine, University of Split, Spinčićeva 1, HR-21000 Split, Croatia; ^2^Department of Gynecologic Oncology, University Hospital Center Zagreb, Petrova 13, HR-10000 Zagreb, Croatia; ^3^Department of Radiotherapy and Oncology, University Hospital Center Rijeka, Krešimirova 42, HR-51000 Rijeka, Croatia; ^4^Department of Medical Oncology, Division of Medical Oncology, University Hospital for Tumors, Sestre Milosrdnice University Hospital Center, Ilica 197, HR-10000 Zagreb, Croatia; ^5^Department of Radiotherapy Oncology, University Hospital Center Osijek, Josipa Huttlera 4, HR-31000 Osijek, Croatia; ^6^Department of Oncology and Nuclear Medicine, University Hospital Center Zagreb, Kišpatićeva 12, HR-10000 Zagreb, Croatia; ^7^Research Unit “Dr. Mirko Grmek”, University Psychiatric Hospital “Sveti Ivan”, Jankomir 11, HR-10.090 Zagreb, Croatia

## Abstract

**Background:**

Although today it is almost preventable, cervical cancer still represents a significant cancer burden, especially in some developing parts of the world. Since the introduction of bevacizumab in the first-line treatment of metastatic disease, improvements of the outcomes were noted. However, results from randomized controlled trials are often hard to recreate in the real-world setting.

**Objective:**

To assess the real-world efficacy and safety of bevacizumab as a first-line treatment of advanced cervical cancer.

**Methods:**

We conducted a retrospective cohort study on the total population of Croatian patients diagnosed with metastatic cervical cancer from 2016 to 2019 who were treated with bevacizumab in combination with cisplatin and paclitaxel (TCB) in the first line. The comparison group was the consecutive sample of patients treated with chemotherapy alone. The primary endpoint was overall survival (OS). Secondary endpoints were progression-free survival (PFS), objective response rate, incidence of adverse events, and the proportion of treatment discontinuation.

**Results:**

We enrolled 67 patients treated with TCB and a control group of 62 patients treated with chemotherapy alone. The TCB cohort had significantly longer unadjusted OS with a median of 27.0 (95% CI 18.5; not calculable) months, compared to 15.5 (10.7; 30.1) months in the chemotherapy-alone cohort. Adjusted OS was not significantly different. PFS was significantly longer for the TCB cohort, with a median of 10.6 (95% CI 8.5; 15.4) months, than for the chemotherapy-alone cohort, with a median of 5.4 (95% CI 3.9; 9.1) months, even after adjustment for baseline covariates (HR_adjusted_ = 0.60; 95% CI 0.39; 0.94; *p*=0.027; false discovery rate <5%).

**Conclusions:**

In a real-world setting, TCB as a first-line treatment of metastatic cervical cancer was associated with longer PFS, better objective disease control rate, and acceptable toxicity profile in comparison to chemotherapy alone. These results may indicate its utility and potential applicability in other parts of the developing world.

## 1. Introduction

Cervical cancer ranks fourth in both cancer incidence and mortality among women, with approximately 604,000 newly diagnosed patients and an estimated 342,000 deaths worldwide in 2020. Furthermore, the burden of cervical cancer is not equally distributed. It is less common and less publicly important in developed parts of the world, whereas it is the most commonly diagnosed cancer as well as the leading cause of death in some developing parts of the world [1]. Even though cervical cancer is almost preventable now, due to primary (HPV vaccine) and secondary (screening programs) prevention currently available, unequal implementation and penetration in the different healthcare systems of countries worldwide could be one of the reasons for the aforementioned global inequality [2, 3]. The association of cervical cancer with lower-income areas in general, the fact that it affects a relatively younger population, the high mortality to incidence ratio, and inadequate implementation of existing prevention altogether make cervical cancer one of the major contributors to the global societal burden. The burden of cervical cancer creates an essential need for international intervention aiming to provide every woman worldwide with an equal chance to prevent and optimally treat this “underserved” disease [1, 4]. Unfortunately, a significant number of patients die, specifically, more than 50% of all newly diagnosed patients per year, underlining the absolute need for therapies with better outcomes [1]. Also, it implies the need for research of novel treatment strategies, such as the tyrosine kinase inhibitors (TKIs) targeting angiogenic kinases, mTOR-inhibitors in PIK3CA mutated cancers, or immunotherapy with checkpoint inhibitors in PD-L1 positive cancers [5]. In contrast to other tumor types, where we have recently witnessed significant improvements in the survival of metastatic patients, there were no significant breakthroughs regarding overall survival in the therapy of cervical cancer since the introduction of platinum and ifosfamide as a standard treatment regimen many years ago [6]. Recently, however, the incorporation of bevacizumab as a part of a first-line therapy option, together with cisplatin and paclitaxel as a chemotherapy backbone, has significantly increased the progression-free survival, response rate, and, most importantly, overall survival rate in metastatic or locally recurrent cervical cancer patient populations [7]. Based on the results of a registrational trial (GOG-240), bevacizumab is accepted as the treatment of choice when coupled with TC chemotherapy in the first-line setting of patients with advanced cervical cancer. Notwithstanding the significant results of the study, randomized controlled trials do not presume the same outcomes in the real-world setting when treating patients [8]. This difference in outcomes is possibly due to the absence of strict inclusion and exclusion criteria and, consequently, population diversity with a higher number of patients with comorbidities in real-world practice. Moreover, the organizational approach to regular work-ups and general oncological care, especially in the underserved parts of the world where the majority of cases are diagnosed, explain the difference between outcomes in real-world settings [9, 10]. Therefore, it is important to monitor the real-world efficacy and safety of the given drug to understand its actual use and benefits in everyday clinical practice [11, 12]. Furthermore, this could be tremendously important for cervical cancer, where the burden of the disease is high in less-developed countries, since bevacizumab is a rather expensive drug. Hence, the aim of this study was to assess the real-world efficacy and safety of bevacizumab as a first-line treatment of advanced cervical cancer in the total population of one of the transitioning countries, namely, Croatia.

## 2. Materials and Methods

### 2.1. Study Design

We conducted a retrospective cohort study on the total population of patients diagnosed with metastatic cervical cancer between 2016 and 2020 in all Croatian oncology centers who were treated with bevacizumab in combination with cisplatin and paclitaxel backbone chemotherapy (TCB) in first-line therapy since its reimbursement status. The control group was the consecutive sample of patients treated with first-line chemotherapy alone for metastatic disease between 2014 and 2019. We conducted this real-world, multicentric study in six Croatian institutions: University Hospital Center Split, University Hospital Center Zagreb, Sestre Milosrdnice University Hospital Center in Zagreb and their Clinic for Tumors, and University Hospital Centers Rijeka and Osijek. The study was approved by the Ethics Committees of all participating institutions. Informed consent was obtained from all living patients before data collection. The data were anonymized before the analysis, and the study was conducted in accordance with the World Medical Association Declaration of Helsinki of 1975, as revised in 2013 [13]. The study protocol was not preregistered, nor were the data reviewed centrally.

### 2.2. Participants

The targeted population was patients diagnosed with recurrent, locally advanced, and metastatic cervical cancer who were treated with TCB as a first-line setting from 2016 to 2020, starting from the time of reimbursement of bevacizumab in Croatia. We did not select the sample but collected the data on the total population treated with TCB. We selected a consecutive sample of patients from the control population. We enrolled patients who received first-line combination chemotherapy treatment for locally recurrent or metastatic disease. The sampling was stopped when the control sample size reached the size of the population treated with TCB. Because we planned to enroll the entire targeted population, we did not perform a power analysis before the start of the study.

### 2.3. Endpoints

The primary efficacy endpoint was the difference in overall survival (OS), defined as the time in months since treatment initiation to death from any cause. OS data in living patients were censored at the time of the last data collection. The secondary efficacy endpoints were the differences in progression-free survival (PFS), objective response rate, and disease control rate between the two cohorts. PFS was defined as the time in months since the initiation of therapy to the progression of the disease from any cause. PFS data in patients alive with no progression were censored at the time of the last exam. The objective response rate was estimated in compliance with the RECIST version 1.1 criteria as stable or progressive disease and partial or complete response. The disease control rate included complete and partial response and stable disease. Secondary safety endpoints were the incidence of treatment-related haematologic, nonhaematologic, or any adverse events of any grade and of grade 3 or 4 and the proportion of patients whose treatment was discontinued to control the adverse events. We defined the grades of adverse events according to the Common Terminology Criteria for Adverse Events v5.0 [14].

### 2.4. Treatment

Patients were given the new standard line treatment for metastatic cervical cancer: TC chemotherapy protocol, which consisted of cisplatin at a dose of 50 mg per square metre of body surface area plus paclitaxel at a dose of 175 mg/m^2^ and bevacizumab at a dose of 15 mg per kilogram of body weight. The therapy was administered at 21-day intervals until disease progression, unacceptable toxicity, or complete response was noted. The control group of patients received the existing standard treatments for metastatic cervical cancer according to the physician's choice. The most common chemotherapy protocol used was TC with cisplatin at a dose of 50 mg per square metre of body surface area or carboplatin AUC 5 or 6 plus paclitaxel at a dose of 175 mg/m^2^. Other protocols used were the combination of cisplatin at a dose of 100 mg/m^2^ applied on day 1 and 5-fluorouracil at a dose of 1000 mg/m^2^ applied on days 1–5 of every 28-day cycle, the combination of ifosfamide 2000 mg/m^2^ plus cisplatin 75 mg/m^2^ every 21-day cycle, the combination of topotecan at a dose of 0.75 mg/m^2^ on days 1–3 plus paclitaxel at a dose of 175 mg/m^2^ on day 1 of every 21-day cycle, and the combination of cisplatin at a dose of 70 mg/m^2^ applied on day 1 and gemcitabine at a dose of 1250 mg/m^2^ applied on days 1 and 8 of every 21-day cycle.

### 2.5. Statistical Analysis

We performed all analyses in the population of patients who received at least one dose of first-line treatment for metastatic disease. We estimated the median OS and PFS using the Kaplan–Meier method with 95% confidence intervals (CIs). To assess the significance of differences in OS and PFS between the two cohorts, we used a two-sided log-rank test in the bivariable analysis and Cox proportional hazard regression in the multivariable analysis with adjustment for age at diagnosis, histology, ECOG performance status before the introduction of first-line treatment for metastatic disease, previous treatment with chemotherapy, and previous treatment with radiotherapy. We handled ties using the Efron method. To check the proportional hazard assumption, we assessed the consistency of the log HR over time by testing the nonzero slope of the generalized linear regression of the scaled Schoenfeld residuals on row time and on the log-time. We visually inspected the parallelism of log-log survival plots in the two cohorts. We calculated the significance of the differences between the two study groups in the objective response rate and safety outcomes using the chi-square (Χ^2^) test. In the analysis of safety endpoints, we calculated relative risk with 95% CIs, and in the multivariable analysis we adjusted the relative risks for the treatment duration and for the aforementioned covariates using a Poisson regression with a robust variance estimator. We declared all missing data below the tables, and we did only the available cases analysis (“pairwise deletion”) although we had no proof that the data were missing completely at random. We did not use any imputation method because the number of missing data was relatively low. We set two-tailed statistical significance at *p* < 0.05 and calculated all CIs at the 95% level. We controlled the false positive rate using the Benjamini–Hochberg procedure with the false discovery rate set in advance at FDR < 5%. We performed the statistical data analysis using StataCorp 2019 (Stata Statistical Software: Release 16. College Station, TX: StataCorp LLC).

## 3. Results

We enrolled 67 patients diagnosed with metastatic cervical cancer who were treated with TCB in the first-line setting and 62 who were treated with chemotherapy alone. The two cohorts were of comparable age (Table 1), menopausal status, body mass index, and no previous therapy for local disease. However, the TCB cohort had a markedly better ECOG performance status before the initiation of first-line treatment for metastatic disease and less often had squamous cervical cancer and previous treatment with chemoradiotherapy and more often surgery alone and surgery + radiotherapy ± chemotherapy as the previous treatment. The duration of the first-line treatment of metastatic disease was somewhat longer in the TCB cohort. Overall, the median follow-up was 14.5 (interquartile range; IQR 8.6–20.5) months in the TCB cohort and 10.9 (3.9–26.4) months in the chemotherapy-alone cohort. The longest follow-up in the last recruited patients was 43.6 months in the TCB cohort and 50.1 months in the chemotherapy-alone cohort.

### 3.1. Efficacy Endpoints

**Figure 1:**
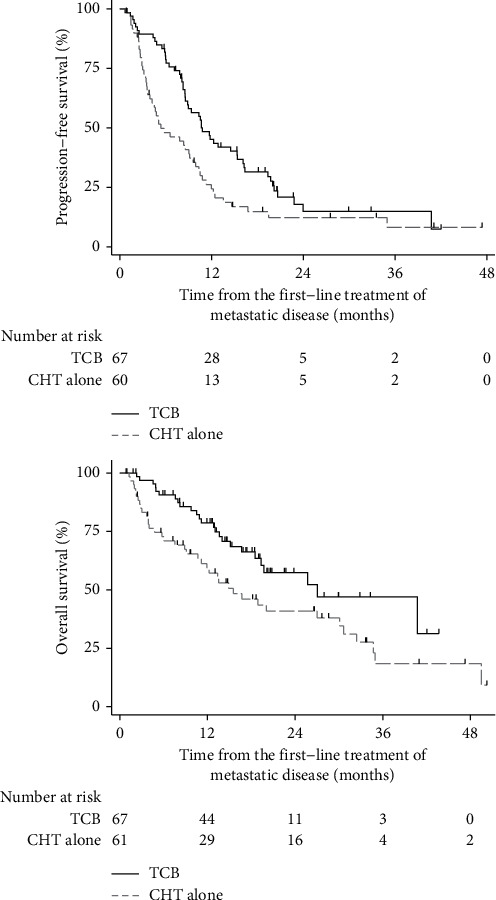
Kaplan–Meier curves of progression-free survival and overall survival from the introduction of first-line treatment for metastatic cervical cancer; TCB = bevacizumab in combination with cisplatin and paclitaxel backbone chemotherapy; CHT = chemotherapy.

The median OS was 27.0 (IQR 18.5-not calculable) months in patients treated with TCB and 15.5 (IQR 10.7–30.1) months in the chemotherapy-only cohort (Table 2; Figure 1). This difference was statistically significant (log-rank test; Χ^2^ = 5.05; *p*=0.025; FDR < 5%). The unadjusted hazard ratio for death, with the chemotherapy-only cohort as the reference cohort, was HR = 0.56 (95% CI 0.34 to 0.93); *p*=0.027; FDR < 5%. After adjustment for age at diagnosis, histology, ECOG performance status before the introduction of first-line treatment for metastatic disease, previous treatment with chemotherapy, and previous treatment with radiotherapy, the hazard ratio for death was no longer significant: HR = 0.78 (95% CI 0.44 to 1.38); *p*=0.389; FDR > 5%. The median PFS from the initiation of first-line treatment for metastatic disease was 10.6 (95% CI 8.5; 15.4) months in the TCB cohort and 5.4 (95% CI 3.9 to 9.1) months in the chemotherapy-only cohort (log-rank test, Χ^2^ = 6.54; *p*=0.011; FDR < 5%) (Table 2, Figure 1). The unadjusted HR was 0.60 (95% CI 0.41 to 0.89; *p*=0.011; FDR < 5%), and the adjusted HR was 0.60 (95% CI 0.39 to 0.94; *p*=0.027; FDR < 5%). Objective response rate, including complete response and partial response, was not significantly higher in the cohort treated with TCB, 38/67 (57%), than in the cohort treated with chemotherapy alone, 24/59 (41%) (Chi-square test; Χ^2^ (1) = 3.23; *p*=0.072; FDR > 5%). The disease control rate, including complete response, partial response, and stable disease, was significantly higher in TCB cohort, 52/67 (78%), than in the chemotherapy-alone cohort, 30/59 (51%) (Chi-square test; Χ^2^ (1) = 9.89; *p*=0.002; FDR < 5%).

### 3.2. Safety Endpoints

The proportion of patients whose treatment was discontinued because of toxicity was not significantly different between the two cohorts (Table 2). Treatment discontinuation was experienced by 11/59 (19%) patients treated with TCB and 9/55 (16%) patients treated with chemotherapy alone (Chi-square test; Χ^2^ (1) = 0.10; *p*=0.749; FDR > 5%). Patients treated with TCB had significantly lower risk for treatment-related haematologic adverse events (RR = 0.60; 95% CI 0.40; 0.90; *p*=0.007; FDR < 5%). The relative risk remained significant after the adjustment for age at diagnosis, histology, ECOG performance status before the introduction of first-line treatment for metastatic disease, previous treatment with chemotherapy, previous treatment with radiotherapy, and duration of the first-line treatment of metastatic disease using TCB or chemotherapy alone (adjusted RR = 0.51; 95% CI 0.32; 0.81; *p*=0.004; FDR < 5%). The risk for nonhaematologic treatment-related adverse events was not significantly different between the two cohorts (Table 2).

## 4. Discussion

The discrepancy in the numbers of diagnosed and successfully treated patients regarding the human development index (HDI) of countries, as well as within countries between developed and less-developed areas, puts cervical cancer patients in a rather “underserved” position [4, 15]. This is also supported by the disparity in research funding among different cancer types. For instance, the parallel can be drawn with breast cancer, which is the most common cancer diagnosed among women. Breast cancer is 4 times more prevalent than cervical cancer but only contributes two times higher to cancer mortality, most likely due to the more than seven times higher research funding investment with a consequently significantly higher number of multiple treatment modalities [1, 16, 17]. Additionally, recent social network analysis has shown that breast cancer is the most frequent keyword used, representing 15% among all keywords, while cervical cancer is used only 2% of the time [18]. It is evident, from the aforementioned information about cervical cancer, that further efforts are needed in the promotion of primary and secondary prevention. Furthermore, enhancement of the existing treatment modalities is needed especially in the second-line setting considering that there is no standard treatment established and that the outcomes are still rather poor and such patients should be considered early for clinical trials regarding novel treatment strategies [19]. However, several significant improvements have been made considering the treatment of locally advanced disease as well as the treatment of metastatic disease with the application of TCB [7, 20]. Since the introduction of bevacizumab as a first-line treatment and the significant improvement in median OS by 3.7 months (HR 0.71), several studies have been conducted to assess its efficacy and safety in the real-world setting [7]. Among the first ones were studies conducted in Spain, Argentina, and British Columbia, and although all of them have shown outcomes from real-world bevacizumab similar to those from the registrational trial, there was no control group. The previous studies were also conducted on a relatively small number of patients from single institutions and with a short period of median follow-up [21–23]. Recently, three studies were conducted in three different centers in China on a larger number of patients and with a control group. Two of these studies had similar results and toxicity profiles to the registrational trial [24, 25]. Meanwhile, the primary outcome of the third study was to assess the toxicity rate, and despite the benefit of bevacizumab, the combined treatment was not well tolerated due to higher grades of neutropenia, gastrointestinal fistula, and hypertension [26]. It is important to emphasize here that all three studies were performed in single institutions, leading to the potential bias of single institution quality of care on the presented outcomes. The results at the national level, with all patients treated included, define the “real” real-world evidence.

Our results in the total Croatian population showed significantly higher PFS and OS among patients treated with TCB in comparison to the control group treated with chemotherapy only. Furthermore, our results have shown higher PFS and OS in comparison to the mentioned studies, in both their length and improvements such as considering control groups. Additionally, the toxicity profile of TCB in our patients was closest to the one from GOG-240, meaning that there was a higher incidence of hypertension and neutropenia, but it did not affect the treatment course or require significant therapy discontinuations.

Considering the costs of bevacizumab treatment, the question arises about its cost-effectiveness for application in everyday clinical practice, especially in challenging financial medical environments where many cases are diagnosed. Our real-world study defines bevacizumab efficacy benefits similar to or above those from the registrational trial. Taking into account that results from randomized phase III trials are often difficult to repeat in general everyday clinical practice, strong recommendations should be made for all new drugs and treatments to be reviewed regarding their clinical benefit in terms of retrospective analysis in different setups, preferably on the country level and within different healthcare systems [8]. Recently, the loss of patent rights for bevacizumab (Avastin) has led to a significantly reduced price of the drug and thus better affordability in many healthcare systems. Our study, together with other real-world studies and the registrational trial, defines the true clinical significance for bevacizumab in the therapy of recurrent or metastatic cervical cancer. While cost-benefit analysis in a developing world was questionable with rather high price of bevacizumab, recent loss of patent rights can, and most probably will, make this treatment more affordable to many underserved patients with recurrent or metastatic cervical cancer. Furthermore, estimation of the “Years of Life Lost” considering that cervical cancer affects a relatively younger population defines even more cervical cancer as underfunded and with absolute need for better and more affordable treatments [27, 28]. Hence, in addition to investment in primary and secondary prevention, investment in affordable treatments with significant clinical benefit should be supported on many different levels and should be given to otherwise underserved patients in many countries around the world.

### 4.1. Limitations of the Study

The first limitation of our study was the lack of randomization into two study groups. For this reason, we cannot reliably rule out the effects of different unmeasured confounders, and the internal validity of our findings is lower than that in randomized controlled trials. At the same time, the real-world setting is the main strength of our study and the cornerstone of its generalizability to real-life populations. The second limitation was the larger number of missing data points for some variables. Data are routinely collected with different levels of rigor, reliability, and precision, and we could not control the basic qualities of electronic records. To minimize the negative effects of these limitations, we carefully collected all the data, checked for their inconsistencies, and cross-checked the suspicious entries in different records. The third limitation was the difference in the proportion of missing data between the two cohorts, although this difference was not large. We had more missing data in the cohort treated with chemotherapy alone than in the cohort treated with TCB. This was partially due to the different regulatory requirements for the recommendation of bevacizumab and other therapies and, consequently, the different levels of comprehensiveness of routinely collected data for patients treated with these two regimens. The fourth limitation was that we selected a consecutive and not the random sample of patients from the control population treated with chemotherapy alone; this could increase the risk of selection bias, but we cannot speculate about the direction or magnitude of the so-caused bias.

## 5. Conclusions

In the real-world setting, bevacizumab utilized as a first-line treatment for metastatic cervical cancer was associated with longer OS and PFS, a better objective disease control rate, and a similar toxicity profile to chemotherapy alone. These results may indicate its utility and potential cost-effectiveness in other parts of the developing world.

## Figures and Tables

**Table 1:** Characteristics of patients and treatment.

	Chemotherapy + bevacizumab (*n* = 67)	Chemotherapy alone (*n* = 62)
Age at diagnosis (years), median IQR)	51 (45–60)	56 (46–61)
Menopause, *n* (%)	45 (67)	38 (61)
Histology, *n* (%)		
Squamous	46 (69)	51 (82)
Adenocarcinoma	16 (24)	7 (11)
Other	5 (8)	4 (6)
ECOG performance status, *n* (%)^a^		
0	38 (57)	15 (25)
1	24 (36)	29 (48)
2	5 (7)	16 (27)
Body mass index (kg/m^2^), median (IQR)^b^	24 (22–29)	24 (22–27)
Previous treatment, *n* (%)		
No therapy before	10 (15)	9 (15)
Surgery alone	10 (15)	1 (2)
Chemoradiotherapy	31 (46)	41 (66)
Surgery + radiotherapy ± chemotherapy	16 (24)	11 (18)
Duration of targeted treatment (months), median (IQR)	4.3 (2.8–8.0)	3.7 (2.0–5.5)
Number of cycles, median (IQR)^c^	6 (5–11)	6 (3–6)
Follow-up (months), median (IQR)	14.5 (8.6–20.5)	10.9 (3.9–26.4)

IQR = interquartile range (range between the 25th and 75th percentiles). ^a^ECOG performance status was missing for 2/62 (3%) patients treated with chemotherapy alone. ^b^Body mass index was missing for 1/67 (1%) patients treated with bevacizumab and for 5/62 (8%) patients treated with chemotherapy alone. ^c^Number of cycles was missing for 3/62 (5%) patients treated with chemotherapy alone.

**Table 2:** Efficacy and safety assessment.

	Chemotherapy + bevacizumab (*n* = 67)	Chemotherapy alone (*n* = 62)	*p*
*Efficacy endpoints*			
PFS (months), median (95% CI)	10.6 (8.5; 15.4)	5.4 (3.9; 9.1)	0.011^*∗*^
Unadjusted HR (95% CI)	0.60 (0.41; 0.89)	1.00 Referent	0.011^*∗*^
Adjusted HR (95% CI)^a^	0.60 (0.39; 0.94)	1.00 Referent	0.027^*∗*^
OS (months), median (95% CI)	27.0 (18.5; n.c.)	15.5 (10.7; 30.1)	0.025^*∗*^
Unadjusted HR (95% CI)	0.56 (0.34; 0.93)	1.00 Referent	0.027^*∗*^
Adjusted HR (95% CI)^a^	0.78 (0.44; 1.38)	1.00 Referent	0.389
Objective response, *n* (%)^b^			
Complete response (CR)	12 (18)	11 (19)	0.013^*∗*^
Partial response (PR)	26 (39)	13 (22)	
Stable disease (SD)	14 (21)	6 (10)	
Progressive disease (PD)	13 (19)	21 (36)	
Could not be determined	2 (3)	8 (14)	
Objective response rate, *n* (%)^b^	38 (57)	24 (41)	0.072
Disease control rate, *n* (%)^c^	52 (78)	30 (51)	0.002^*∗*^

*Safety endpoints*			
Treatment discontinuation because of toxicity, *n* (%)^d^	11 (19)	9 (16)	0.749
Treatment-related adverse events			
Any grade	54 (81)	53 (85)	0.461
Grades III-IV	33 (49)	41 (66)	0.053
Treatment-related, haematologic adverse events			
Any grade	44 (66)	53 (85)	0.009^*∗*^
Grades III-IV	18 (27)	31 (50)	0.007^*∗*^
Treatment-related, nonhaematologic adverse events			
Any grade	51 (76)	50 (81)	0.533
Grades III-IV	22 (33)	21 (34)	0.901

CI = confidence interval; PFS = progression-free survival; OS = overall survival; HR = hazard ratio; n.c. = not calculable. ^a^Analysis was adjusted for age at diagnosis, histology, ECOG performance status before the introduction of first-line treatment for metastatic disease, previous treatment with chemotherapy, and previous treatment with radiotherapy. ^b^Objective response rate includes complete and partial response; data were missing for 6/67 (10%) patients treated with TCB and 3/62 (5%) patients treated with chemotherapy alone. ^c^Disease control rate includes complete and partial response and stable disease. ^d^Data on treatment discontinuation because of toxicity were missing in 8 (12%) patients treated with TCB and 7 (11%) patients treated with chemotherapy alone. ^*∗*^False discovery rate <5%.

## Data Availability

The data used to support the findings of this study are available from the corresponding author upon request.
